# Polychaete Community of a Marine Protected Area along the West Coast of India—Prior and Post the Tropical Cyclone, *Phyan*

**DOI:** 10.1371/journal.pone.0159368

**Published:** 2016-08-24

**Authors:** Soniya Sukumaran, Tejal Vijapure, Priti Kubal, Jyoti Mulik, M. A. Rokade, Shailesh Salvi, Jubin Thomas, V. S. Naidu

**Affiliations:** Regional Centre,CSIR-National Institute of Oceanography, Mumbai, Maharashtra, India; Universita degli Studi di Genova, ITALY

## Abstract

Tropical cyclones are extreme random meteorological events that can have profound implications to coastal biodiversities. Given that the frequency, intensity and duration of these events are poised to increase due to the global climate change, understanding the ecological impacts of such erratic occurrences becomes imperative to devise better management strategies. The eventful passage of the tropical cyclone, *Phyan*, along the northwestern coast of India in November 2009, coupled with the availability of historical data presented a rare opportunity to elucidate the consequences on the polychaete assemblages of the Malvan Marine Sanctuary and their subsequent recovery. This was achieved by comparison of the pre- and post-*Phyan* seasonal data from four different sites in and around the Sanctuary. MDS analyses and polychaete community parameters suggested conspicuous cyclone related effects on the polychaete community characteristics in the three outer stations off Malvan, whereas the relatively protected bay station remained more or less unscathed. Impacts, attributable to the cyclone apart from seasonal variations, included changes in polychaete composition, reductions in total polychaete density, species diversity, evenness and functional groups. Dominance of the opportunistic polychaete, *Paraprionospiopatiens* was all pervasive just after *Phyan*, resulting in poor diversity and evenness values. In the outer stations, diverse feeding modes present prior to the cyclone were replaced by microphagous feeders post the disturbance. However, the study also observed complete recovery as substantiated by the improvement inpolychaete density, diversity indices and re-instatement of multiple feeding guilds in affected areas. This resilience of the coastal waters off Malvan is attributed to its marine protected status, implying that reduced human interference aided rapid revival of damaged ecosystems.

## 1. Introduction

Tropical cyclones can be destructive, often leading to extensive damage to coastal zones, communities and ecosystems especially in the Indian Ocean rim countries[1].Extremely powerful cyclonesare especially significant in shaping benthic communities, having the potential to cause severe damage to benthic fauna over large areas. Scouring, re-suspension, deposition or erosion of seafloor sediments associated with high wave action and currents [2] as well as sudden salinity fluctuations associated with high-intensity tropical cyclones can cause high mortality and redistribution of macrobenthos[3,4].Changes in sediment granulometry subsequent to cyclones and tsunamis have been shownto have modified thecommunity composition and structure of resident benthic organisms[5–6]. Defaunation resulting from such episodic perturbations, often leads to recolonisation by different functional groups,thereby creating shifts in benthicstructure[3]. Investigations on the ecological damages of such natural sporadic events on coastal benthic assemblages and their subsequent recovery assume considerable significance particularly because of the predicted increase in the frequency, duration and intensity of tropical cyclones as a consequence of mounting greenhouse gases or regional climate shifts [1, 7–9].So far, studies on the impacts of cyclonic events on benthic communities have been largely focused on intertidal/beach habitats due to their higher vulnerability to cyclone- / tsunami-induced disturbances than the subtidal zones [10]. In such an assessment of post-incident ecological modifications, the pre-impact datasets have a vital role for reliable evaluation which have been missing in several studies available in the literature.The present study compares the benthic ecological modifications induced due to the tropical cyclone, *Phyan*, at Malvan on the northwest coast of India which was being routinely monitored by us prior to the cyclone, providing an excellent opportunity for the post-cyclone assessment.

The tropical cyclone, *Phyan*, which developed over the south-eastern Arabian Sea during 9–12 November 2009, swept northward along the eastern Arabian Sea and finally made its landfall on the northwestern coast of India leaving a trail of large scale destruction of crops, property and loss of lives in its wake making it the second biggest natural calamity to hit the Indian coast after the December 2004 Indian Ocean Tsunami [11]. *Phyan*hit Malvan, on the northwest coast of India on 11 November 2009 and lasted for almost 6 hours wreaking havoc on the entire coastline due to intense sea surface waves and increased coastal surges [11,12]. The central pressure and maximum sustained surface speed was estimated to be 988 hPa and 83 km/hr respectively during 11 November 2009 [1].

Sustained monitoring of coastal ecosystems tounderstand natural and anthropogenic impacts is a prerequisite for tropical countries that are often sites of high coastal and marine biological diversity [13]. Despite the rich biodiversity of the Indian coast, ecological studies dealing with the impact of natural perturbations on the coastal biodiversity and subsequent recovery have received scant attention. Possible reasons could be the absence of reliable pre-event baselines based on which the impact of the natural disasterscould be gauged and subsequent recovery can be understood in proper perspective[14] and the erratic nature of such events. The Coastal Ocean Monitoring and Prediction System (COMAPS), a long term monitoring programme funded by the Ministry of Earth Sciences (MoES), Government of India, covers critical locations along the 7500 km long Indian coast line including Malvan, a well known biodiversity hot spot, particularly thronged by scuba enthusiasts for its beautiful coral formations and associated rich marine life. The availability of pre-cyclone ecological data for Malvan presented a unique opportunity to evaluatethe consequences of a tropical cyclone on the benthic ecosystem of a coralline marine protected area. A perusal of published literature on the impacts of *Phyan* onthe coastal zone indicates that most observations were on the physical responses [1, 11, 15]with very little information on the biological disturbances and subsequent recovery[1].

Polychaetes are the major constituents of macrobenthos[16], ubiquitous, have restricted movements with diverse trophic guildsand reproductive strategies whichare capable of differential response to natural or anthropogenic disturbances, rendering the group useful biological indicator in bio-monitoring studies [17]. Polychaetesare consideredto be excellent indicators of environmental disturbances[18–19] and are often the pioneering benthic groups that re-colonize after disturbances [20]. With pre-cyclone time-series datasets Malvan which is a declared Marine National Sanctuary provides such an opportunity to investigate the post-cyclone ecological changes in the benthic assemblages. Since this can be a mammoth exercise both in terms of time and man-days, the indicator taxon, Polychaetais considered in this investigationto represent the macrobenthicspecies richness of the study area.

Thus, the data collected immediately after *Phyan* (Postmonsoon2009) arecompared with the pre-cyclonerecords (Premonsoonand Postmonsoon 2007) to gauge the changes in polychaeteassemblages subsequent to the eventwrought by cyclonic processes. To facilitate sound comparison and elucidate the recovery of polychaetes,the same stations sampled prior to cyclone were covered during Monsoon 2011, Postmonsoon 2011 and Premonsoon 2012. The study spanning over asix year period, were planned to also account for the seasonal natural variations in the benthic patterns. The specific aims of the present study were (1) to evaluate the immediate impact of the cyclone on the subtidalpolychaete assemblages ofthe sensitive biodiverse Malvan area; and (2) to track the recovery process thereafter.

## 2. Materials and Methods

### 2.1 Study area

**Fig 1:**
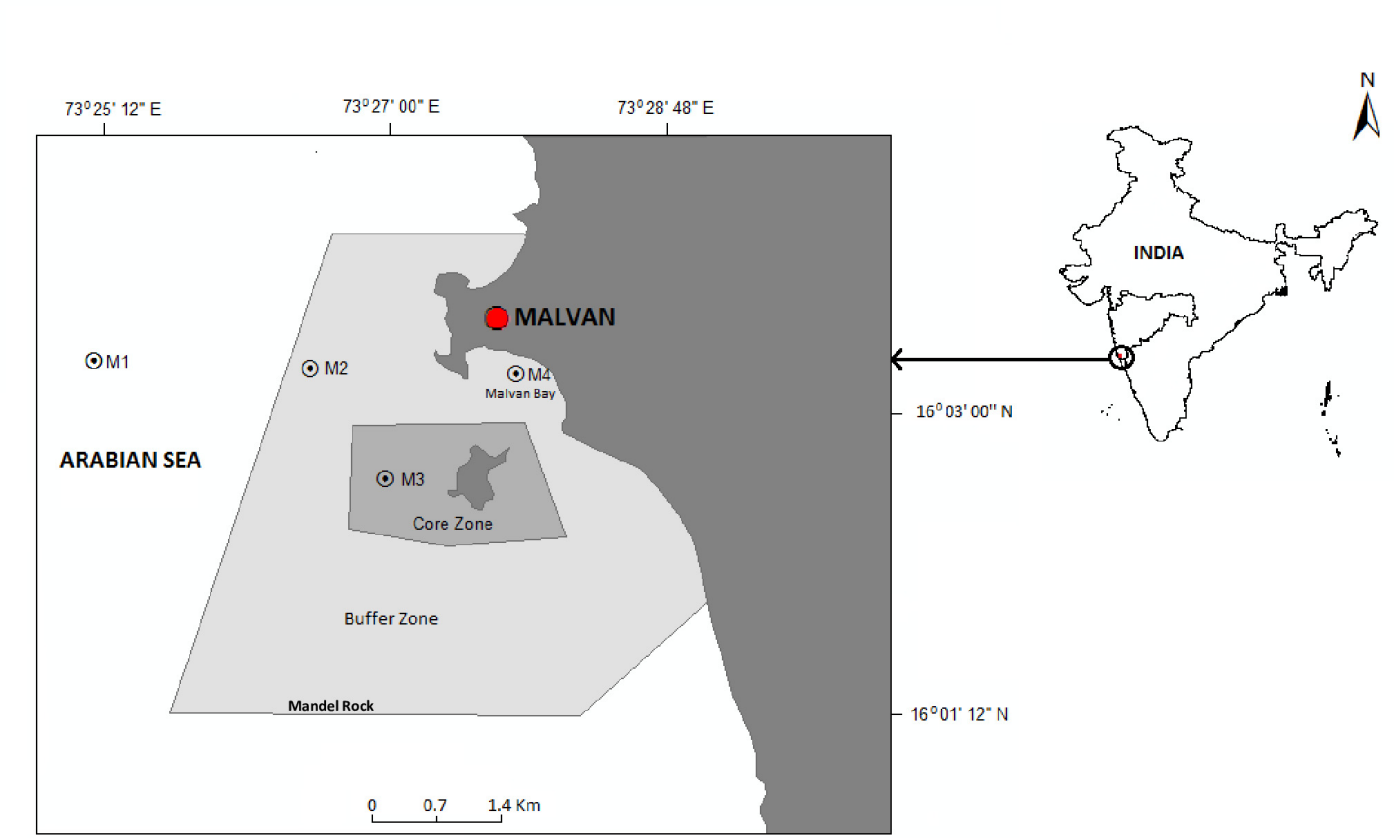
Study area and position of sampling stations. Shaded region denotes the limits of the Malvan Marine Sanctuary.

Malvan coast, which is open to the Arabian Sea and dominated by rocky outcrops with intermittent sandy beaches has been declared by the Government of India a Marine Sanctuaryin 1987 to protect the fragile ecology of this marine hotspot and to buffer it from the high fishing activity and rapid urbanization of the town. The sanctuary (16°05’ N, 73°30’E), has a core zone of 3.182 km² and a buffer zone of 25.94 km² (total area being 29.122 km²) (Fig 1) with the core zone encompassing the historically important Sindhudurg fort and other submerged rocky structures around. The north eastern border of the buffer zone is 50 m from the Malvanportwhere high fishing activities are common, on the eastthere is a semi-circular sandy beach and its southern boundary is near the Mandel rock. (www.icsf.net).Theweather is typical of the central west coast of India with premonsoon seasonof oppressive heat from March to May followed by the southwest monsoon season from June to September-the period of heavy precipitation;October to January form the postmonsoon season of relative pleasant weather. The Malvan region is under the considerable influence of semi-diurnal tides with the mean spring and neap tidal ranges of 2.4 and 1.0 m respectively.

Sampling was conducted at four stations off Malvan, which were the part of the ongoing monitoring programme under COMAPS referred earlier (Fig 1). Station M1 (16°03.215’N; 73°25.117’E; 18 m depth) was the outermost station located outside the boundary of the Marine Sanctuary. Station M2 (16°03.333’N; 73°26.449’E; 15 m depth) was in the buffer zone of the Marine Sanctuary andStation M3 (16°02.857’N; 73°27.333’E; 15 m depth) was in the core zone. Station M4 (16°03.165’N; 73°27.836’E; 1 m depth) was in the Malvan Bay and consequently nearest to the sandy shore.Stations M1, M2and M3 situated in the open sea were termed as the ‘outer stations’ whereas the station M4, that was located in the Malvan Bay, was termed as the ‘bay station’.

### 2.2 Sampling and Analyses

**Fig 2:**
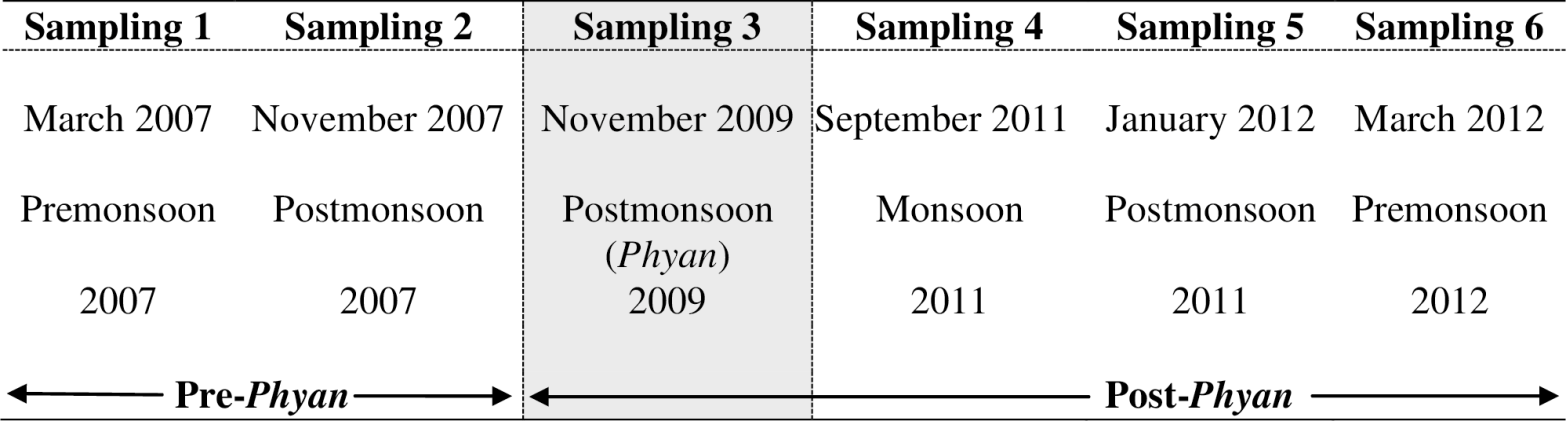
Timeline showing six sampling periods, nomenclature and classification.

Permissions were obtained from Maharashtra Maritime Board and Marine Police, Malvan for the field surveys. The study did not involve any endangered or protected species. Pre-*Phyan* sampling was conducted at all four stations detailed above during March 2007 (hereafter Premonsoon 2007) and November 2007 (hereafter Postmonsoon 2007). The same stations were sampled on 19 November 2009 (7 days after the cessation of *Phyan*) and resultant data tagged as Phyan 2009 would represent the ecological status of the study area immediately after the cyclone. Post-*Phyan*sampling at the same four stations during September 2011 (hereafter Monsoon 2011), January 2012 (hereafter Postmonsoon 2011) and March 2012 (hereafter Premonsoon 2012) provided the information on the temporal recovery of the polychaete assemblages.The details of sampling schedule are given in Fig 2. Station M3 could not be sampled during Premonsoon 2007 due to technical reasons.Similar sampling strategy,as detailed below, was employed for all four stations during the six sampling events to generate comparable data. Near bottom water samples were collected using a Niskin water sampler which were then analysed for pH, salinity and dissolved oxygen (DO) following the standard methods [21]. Four replicate sediment samples were taken at each station during every sampling eventusing a 0.04 m^2^ van Veengrab for macrobenthic analyses. A grab sample was retrieved separately for analyzing sediment texture [22] and organic carbon [23]. The sediments were sieved through a 0.5 mm mesh sieve and the retained residual matter was fixed in 5% buffered formaldehyde mixed with Rose Bengal. The dominant taxa, Polychaeta, were then sorted and identified to the lowest possible taxon, enumerated, averaged across replicates at each station and expressed as number of individuals per square meter (ind.m^-2^). Post identification, polychaete species were assigned to their feeding guilds (Microphages, macrophages, macrophage-microphages, macrophage-omnivores, omnivores) following Jumars et al.[24].

### 2.3 Data Analyses

To assess the impact of cyclone and the subsequent recovery of benthic assemblages, changes in the polychaete community structure before and after *Phyan* were analysed by univariate, multivariate and graphical methods. The macrobenthic community status in the study area was evaluated by univariate analyses of polychaetespecies density data andby comparing the number of individuals (N).Species richness was estimated by theMargalefindex (*d*) andHurlbert's index (*ES*_100_)while the evenness measures included the Pielou’sevenness (*J’*) and Simpson's index (1-λ′). The Shannon Wiener index (*H’log*_*2*_) was calculated to compare the species diversity across locations and seasons. Multivariate analyses included the ordination of fourth root transformed polychaetespecies density data using the Bray-Curtis similarities by non-metric Multi-Dimensional Scaling (MDS) to visualize the dissimilarity in polychaete community structure spatially and temporally. Univariate and multivariate analyses were carried out using the statistical software, PRIMER v6.The relative proportion of trophic groupsduring the six sampling periods was plotted in graphs using Excel 2007 for comparison of polychaete feeding guild compositionbefore and after *Phyan*.

Two-way analyses (*Impact* x *location*) were undertaken by employing the Permutational Multivariate Analyses of Variance (PERMANOVA)[25]within the PRIMER software package to examine the changes in polychaete structure due to *Phyan*. PERMANOVA permits testing of complex multifactorial designs based on similarity matrices of polychaete species data, without the constraints of conventional parametric testing and yieldsFischer’s *F*-statistic with *p*-values obtained by permutation. The sampling design included two factors: *Impact*with 2 levels; *Before Phyan*and *After Phyan*and *Location* which included the 4 stations (i.e. 4 levels).Pair-wise tests were used to determine the significantdifferences in the structure of the polychaete assemblage among the four locations. The Spearman Rank correlation coefficient (*r*) was estimated between the polychaeteunivariate indices, polychaete feeding groups, density of dominant polychaete taxa and the environmental parameters using Statistica 7.

Polychaetespecies data was initially subjectedto the Detrended Correspondence Analysis (DCA) [26] to determine the model (linear or unimodal) of numerical techniques. As the gradient length was more than 2SD, Canonical Correspondence Analysis (CCA) was selected as the unimodal response model [27–28] toassess the major abiotic parameters responsible for the shift in the polychaete community structure, using CANOCO version 4.5[29]. Analysis included 17 significantly contributing species having over 1% of relative density and 7 selected environmental variables. Monte Carlo Permutation test (with 499 unrestricted permutations) was incorporated to test the significance of the ordination axes.

## 3. Results

### 3.1 Environmental variables

**Fig 3:**
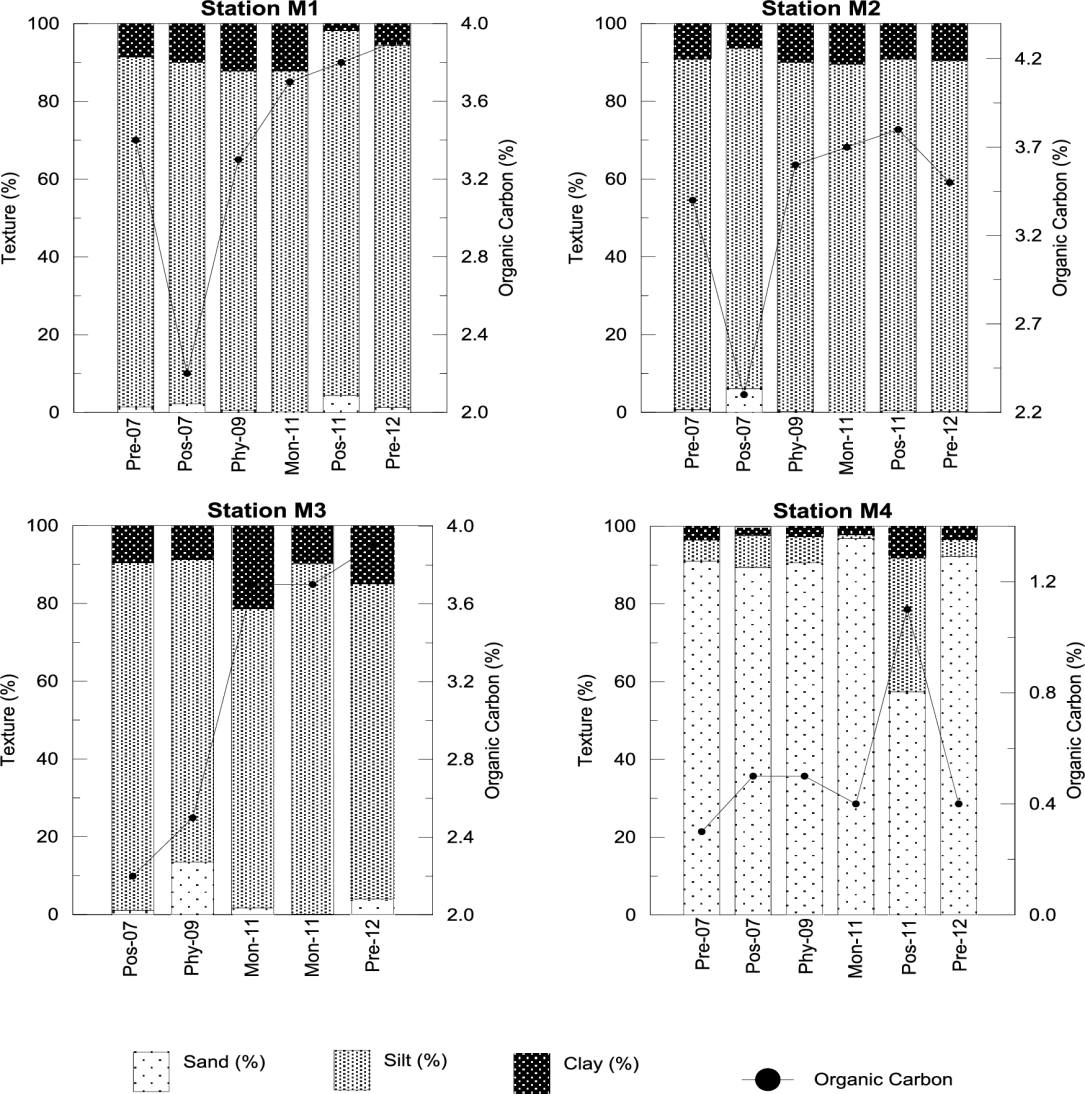
Sediment composition and Organic Carbon at the four stations during the 6 sampling occasions Premonsoon 2007, Postmonsoon 2007, Phyan 2009, Monsoon 2011, Postmonsoon 2011 and Premonsoon 2012.

A comparison of bottom water characteristics across the sampling years is given in Table 1. DO and pH were largely unaffected by the impact of the cyclone.Drop in salinity was minor (33.5±0.3psu) as compared to the corresponding 2007 values (33.9±0.1psu) though there was heavy precipitation, probably due to efficient mixing caused by turbulence.It is evident from Fig 3 that the bottom sediments in the study area were broadly of two types. Stations M1, M2, and M3 had a clayey-silt texture whereas sediments of station M4 were sandy with minor clay and silt fractions. Immediately after *Phyan*, Stations M1 and M2 sustained higher proportions of clay almost obliterating the sand fraction while,station M3 on the contrary, had a higher proportion of sand (13.5%). The sediments at station M4 remained sand dominated before and after *Phyan*. The fine-grained clayey-silt sediments with the organic carbon contents of more than 2% dominated the substrataatstations M1, M2 and M3 whileat station M4, the content was low (0.3–1.1%)as expected for sandy substratum. An increase in the organic content was also evident post *Phyan*in the study area (Fig 3).

**Table 1:** Chemical parameters across study periods.

	Premonsoon 2007	Postmonsoon 2007	Phyan 2009	Monsoon 2011	Postmonsoon 2011	Premonsoon 2012
**DO(mgl**^**-1**^**)**	5.4±1	6.6±0.6	6.5±0.5	6.3±0.5	6.1±0.2	7.1±0.4
**pH**	7.9±0	8.1±0.1	8.2±0.1	8.2±0.2	8.2±0.1	8.3±0
**Salinity(psu)**	35.1±0.6	33.9±0.1	33.5±0.3	32.7±0.3	34.9±0.1	35.7±0.2

### 3.2 Polychaetedensity and diversity

**Fig 4:**
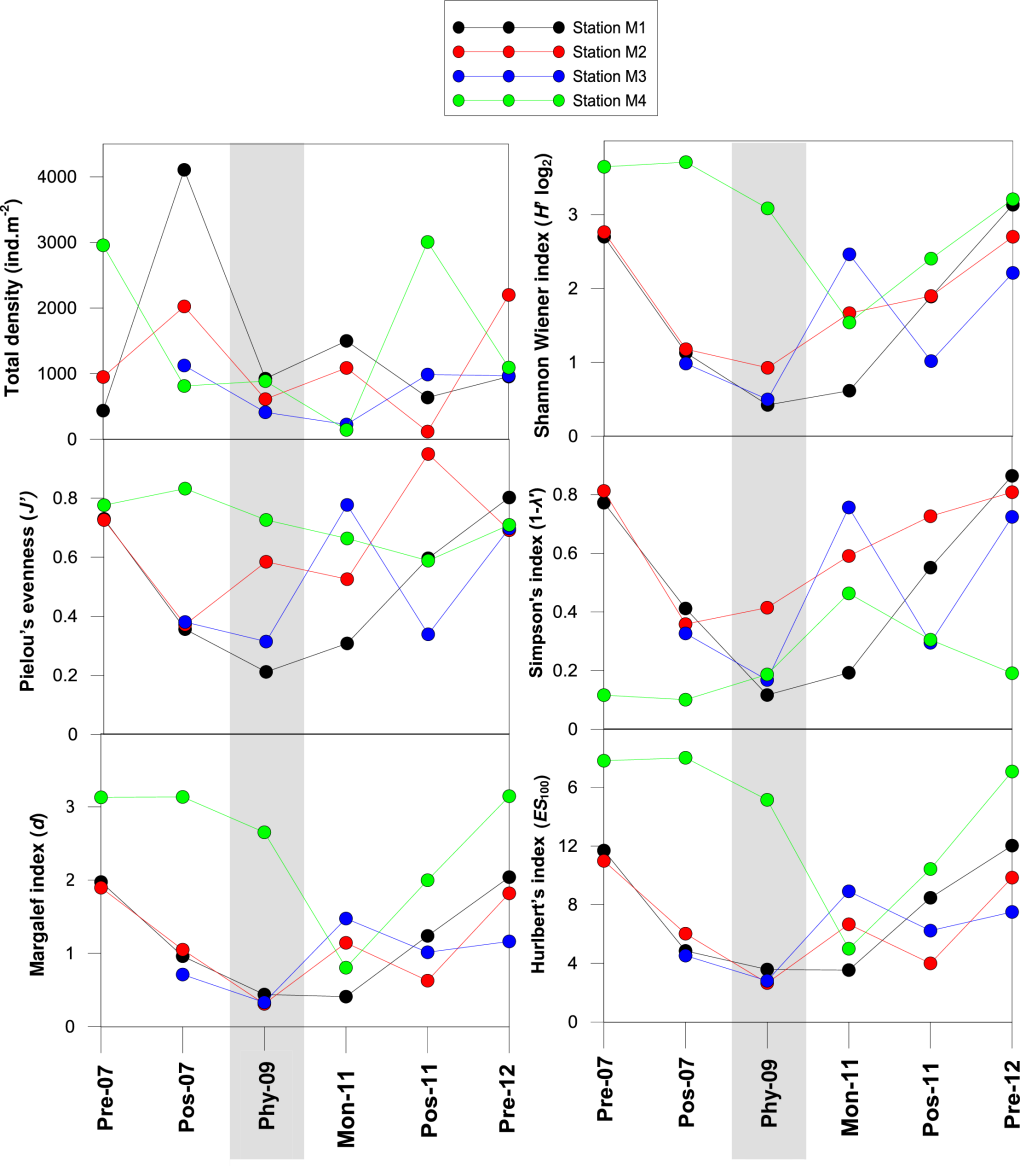
Univariate analyses using polychaete density at the four stations during the 6 sampling occasions: Premonsoon 2007, Postmonsoon 2007, Phyan 2009, Monsoon 2011, Postmonsoon 2011 and Premonsoon 2012. Shaded region denotes sampling immediately after *Phyan*.

Average polychaete density in the outer stations (M1-M3) decreased by 73.1% from Postmonsoon 2007 to Postmonsoon 2009 (*Phyan*) and recovered by 12% by Postmonsoon 2011. On the contrary, an enhancement in average density (9%) occured at the bay station (M4) during Phyan 2009 as compared to the pre-event baseline. Minimum polychaetedensity at the bay station, M4 was during Monsoon 2011 (Fig 4). Overall, 73 polychaetespecies belonging to 32 families were identified across all stations and sampling periods (S1 Table). A progressive reduction in the number of polychaetespecies was observed in the three outer stations during Premonsoon 2007 (n = 20), Postmonsoon 2007 (n = 13) andPhyan 2009 (n = 5). Conversely,post *Phyan*, an increasing trend in species numbers were seen during Monsoon 2011 (n = 15), Postmonsoon 2011 (n = 12) and Premonsoon 2012 (n = 21). The bay station, M4, had least number of species (n = 5) during Monsoon 2011and highest number during Premonsoon 2007 (n = 26) and Premonsoon 2012 (n = 23).

Shannon diversity was lowest during Phyan2009 at the outer stations M1 (*H' log*_*2*_ = 0.4),M2 (*H' log*_*2*_ = 0.9) and M3 (*H' log*_*2*_ = 0.5)(Fig 4) as against the higher values, during Premonsoon 2007 (*H' log*_*2*_ = 2.7±0) and Premonsoon 2012 (*H' log*_*2*_ = 2.7±0.4) at the same locations.The bay station,M4, had high Shannon valuesduring all sampling periods (*H' log*_*2*_ = 2.4–3.7) except during Monsoon 2011 (*H' log*_*2*_ = 1.5). Similarly, the species richness of the outer stations were lowest during Phyan 2009 (*d* = 0.4±0.1; *ES*_100_ = 3.0±0.5) and higher during Premonsoon 2007 (*d* = 2.0±0.1; *ES*_100_ = 11.3±0.5) and Premonsoon 2012 (*d* = 1.7±0.4; *ES*_100_ = 9.8±2.3)(Fig 4). The bay station sustainedhigh species richness during all sampling occasions (*d* = 2.0–3.1; *ES*_100_ = 10.4–18.0) with the exception of Monsoon 2011 (*d* = 0.8; *ES*_100_ = 5.0).All four stations had anevenly proportioned polychaete community structure as indicated by high evenness values (*J'*>0.7; 1-λ′ ≥0.7) during Premonsoon 2007 and 2012. However at the outer stations, dominance by opportunistic taxa was revealedduring the Postmonsoon 2007and 2009 (*Phyan*)based on the low evenness values (*J'*≤0.6; 1-λ′ ≤0.4) (Fig 4) whereas, thebay station displayed consistent evenness values (*J'*>0.6;1-λ′≥0.5).

Prior to the advent of *Phyan*, 24 polychaete species that dwelled at outer stations had dwindled down to mere 5 species just afterthe cyclone before recovering to 30 species overthe extended post cyclone period (S1 Table). The five polychaete taxa present at the outer stations immediately after *Phyan* were Eunicidae (gen. sp.), *Lumbrineris*sp., *Aonidellacirrobranchiata*, *Paraprionospiopatiens* and *Cossuracoasta*.Of the 30 species recordedduringthe post *Phyan*period, only 6 were *come-back species* (those present before *Phyan*).The number of species present at the bay station M4 before, just after and post cyclone were 36, 19 and 31 respectively with only 5 *come-back species*. The most dominant species,*P*. *patiens* which comprised 76% of the polychaetedensity at the outer stations during Postmonsoon 2007 had proliferated to 86% immediately after *Phyan* (Table 2).

**Table 2:** Major species (contribution~ 80%) at outer and bay stations during each sampling event. Numbers in parenthesis indicate species percentage composition to the nearest integer.

	Premonsoon 2007	Postmonsoon 2007	Phyan 2009	Monsoon 2011	Postmonsoon 2011	Premonsoon 2012
**Outer Stations M1-M3**	*Cossura coasta*(22)	*Paraprionospio patiens*(76)	*Paraprionospio patiens*(86)	*Paraprionospio*sp.(62)	*Cossura coasta*(73)	*Cossura coasta*(28)
	Capitellidae (gen.sp.)(18)	*Magelona cincta*(15)	*Cossura coasta*(12)	*Cossura coasta*(28)	*Paraprionospio* sp.(6)	Maldanidae(gen.sp.)(17)
	*Capitella capitata*(16)				*Magelona cincta*(5)	*Heterospio*sp.(14)
	*Mediomastus*sp.(15)				* *	*Sternaspis scutata*(14)
	*Parheteromastus*sp.(9)					*Ninoe*sp.(13)
**Bay Station M4**	*Scoloplos armiger* (24)	*Scoloplos armiger* (19)	*Paraprionospio patiens*(35)	Capitellidae (gen.sp.)(65)	*Paraprionospio Patiens*(51)	*Scoloplos uniramus*(37)
	*Sabellastarte longa* (17)	*Aonidella cirrobranchiata*(15)	*Scoloplos armiger* (17)	*Scoloplos uniramus*(17)	Capitellidae(gen.sp.)(14)	*Eunice pennata*(19)
	*Novafabricia bansei*(10)	*Cirratulus*sp.(12)	*Aonidella cirrobranchiata*(16)		*Scoloplos Uniramus*(13)	*Nematonereis*sp.(7)
	*Cirratulus*sp.(9)	*Novafabricia bansei*(11)	Sabellariidae (gen.sp.)(5)		*Magelona cincta* (6)	*Isolda pulchella*(6)
	*Eunice* sp.(6)	*Eunice* sp.(9)	*Aglaophamus dibranchis* (4)			Capitellidae(gen.sp.)(4)
	Orbiniidae (gen.sp.)(5)	*Sabellastarte longa* (6)	*Glycera longipinnis*(4)			*Micronephthys oculifera*(3)
	Capitellidae (gen.sp.)(5)	Spionidae (gen.sp.)(5)				*Armandia*sp.(3)
	*Boccardia polybranchia*(4)	*Glycera longipinnis*(4)				*Phyllodoce capensis*(3)
	*Glycera longipinnis*(4)	* *				* *
	*Parheteromastus*sp.(4)	* *				* *

**Fig 5:**
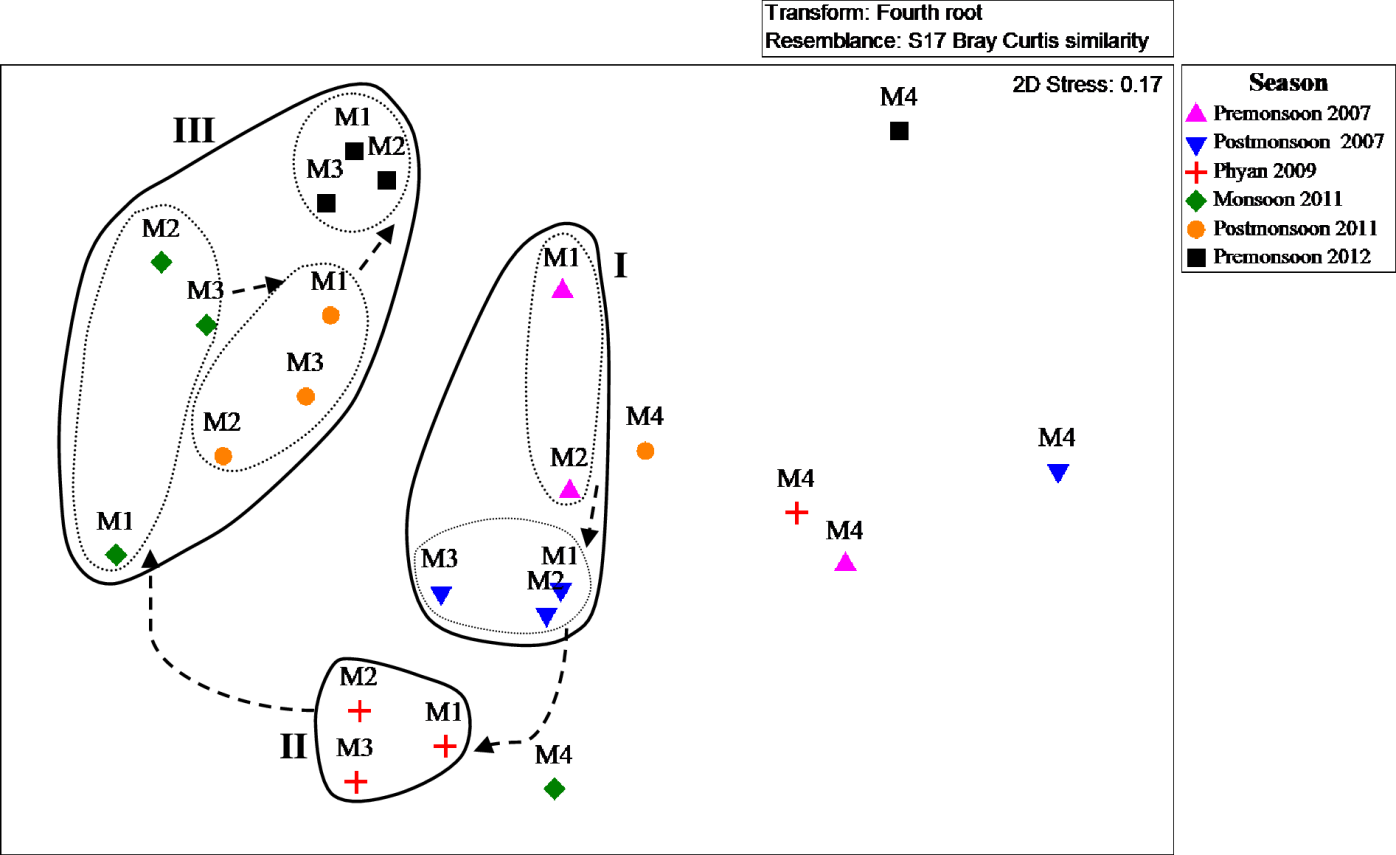
Non-metric MDS ordination of polychaete assemblages at the four stations during the 6 sampling occasions; Premonsoon 2007, Postmonsoon 2007, Phyan 2009, Monsoon 2011, Postmonsoon 2011and Premonsoon 2012.

The segregation between the sand dominated bay station (M4)and the clay-silty outer stations (M1–M3) was clearly demonstrated in the MDS plot (Fig 5). Seasonal variations weremore prominent than spatial variations among the three outer stations as seen from the cyclical distribution pattern of the station clusters in the MDS plot. It was also evidentthat the post-*Phyan* assemblages had a different species composition as compared to the pre-*Phyan* assemblages. Significant shifts in polychaete community structure before and after *Phyan*wereevidenced by PERMANOVA (*F*_*s*_ = 2.1, *p*<0.05). Similarly, PERMANOVA (*F*_*s*_ = 1.6, *p*<0.05) confirmed significant spatial variations inthe polychaete community structure, probably due to the dissimilarity of species composition between the outer and the bay stations as seen in the MDS. Subsequent pair-wise comparisons of the four sampling locations indicated significant variance between the polychaete community structure of each outer station (stations M1-M3) and thebay station, M4 (t = 1.59–1.64, *p*<0.001). Differences between the polychaete communities of the three outer stationswere not significant as per PERMANOVA (t = 0.49–0.87, *p*<0.98).

### 3.3 Polychaete trophic composition

**Fig 6:**
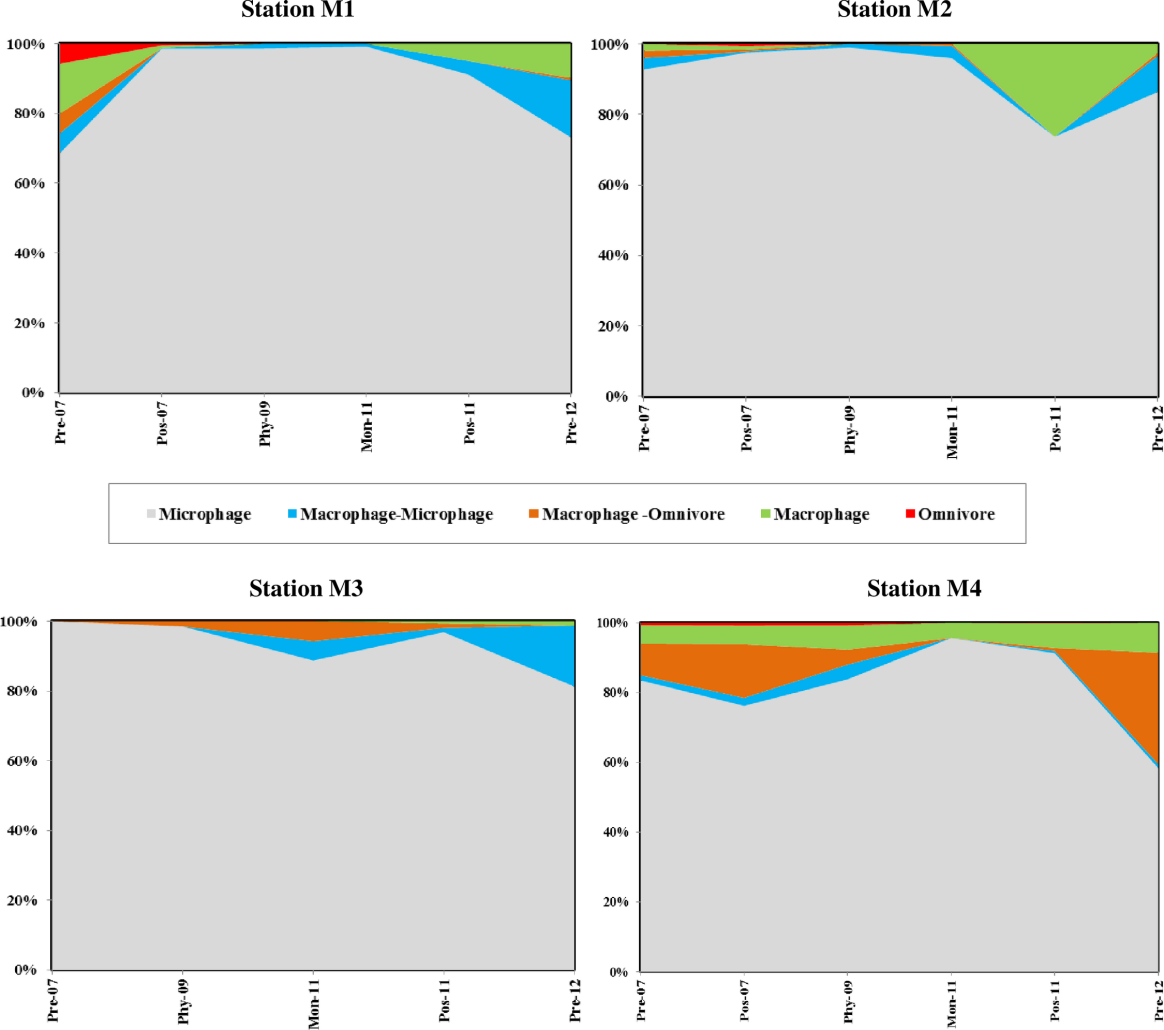
Composition of feeding guilds at the four stations during the 6 sampling occasions: Premonsoon 2007, Postmonsoon 2007, Phyan 2009, Monsoon 2011, Postmonsoon 2011 and Premonsoon 2012.

Microphage feeders were the dominant trophic groups in the outer stations under all conditions (Fig 6). Analyses of feeding guilds at stations M1 and M2 showed a pattern of replacement of diverse feeding types that were present during Premonsoon 2007 with microphages during Postmonsoon 2007. During Phyan2009 and Monsoon 2011, only microphagousfeeders prevailed with a minor representation of macrophage-microphagous feeders. At station M3, macrophage-microphages appeared and macrophage-omnivores increased post *Phyan* displacing the microphagous feeders to some extent.It is also evident from Fig 6 that the most trophicallybalanced polychaete assemblages at the outer stations were present during post *Phyan*(Premonsoon 2012).At the station M4, the polychaete assemblages were well represented by all five feeding groups during Phyan 2009. Macrophages were omnipresent during all six sampling seasons and microphagous feeders dominated (>90%) during Monsoon 2011 and Postmonsoon 2011. However, polychaete communities with multiple feeding groups were re-established in the bay by Postmonsoon2011 (Fig 6).

### 3.4 Correlations between polychaete and environmental variables

**Fig 7:**
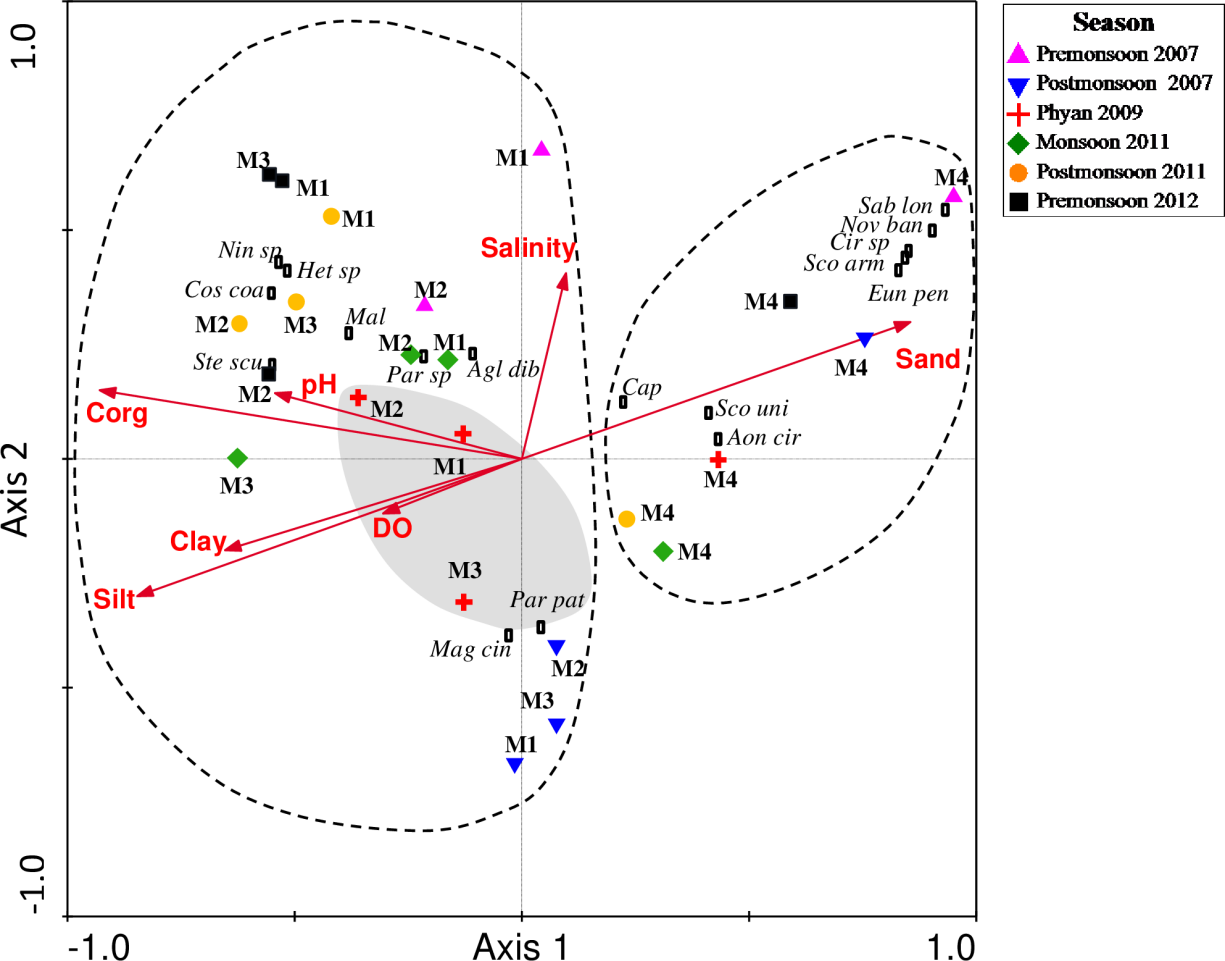
Canonical correspondence analyses triplot of 17 most abundant polychaete species and 7 explanatory variables at the four stations during the 6 sampling occasions; Premonsoon 2007, Postmonsoon 2007, Phyan 2009, Monsoon 2011, Postmonsoon 2011 and Premonsoon 2012. ●:stations; →:environmental variables; Sand; Silt; Clay; C_org_; Salinity; DO; pH; Δ:Polychaete species;; *Agl dib*: *Aglaophamusdibranchis*; *Eun pen*: *Eunice pennata*; *Nin sp*: *Ninoe*sp; *Aon cir*: *Aonidellacirrobranchiata*; *Par*sp: *Paraprionospio*sp; *Par pat*: *Paraprionospiopatiens*; *Het* sp: *Heterospio*sp; *Mag cin*: *Magelonacincta*; *Cir*sp: *Cirratulus*sp.; *Scouni*: *Scoloplosuniramus*; *Sco arm*: *Scoloplos armiger*; *Cos coa*: *Cossuracoasta*; *Cap*: Capitellidae (gen. sp.); *Mal*: Maldanidae (gen. sp.); *Stescu*: *Sternaspisscutata*; *Sab lon*: *Sabellastarte longa*; *Nov ban*: *Novafabriciabansei*. General groupings of stations and species are indicated. Shaded stations are *Phyan* impacted).

Diversity parameters i.e. number of species (S), Shannon Wiener index(*H’log*_*2*_),Margalef species richness (*d*) and Hurlbert's rarefaction index (*ES*_100_) were positively correlated with sand and salinity but negatively correlated with clay (Table 3). Among the trophic groups, macrophages had a positive correlation with sand and salinity and negative correlation with clay.Macrophage-omnivores were positively correlated with sand and salinity and negatively with clay and organic carbon (Table 3).The first 2 axes of CCA triplot explained 62.7% of the variance of species-environment relationship. Monte Carlo permutation test (with forward selection) indicated that sand was positively correlated whereas silt, clay, organic carbon and pH were negatively correlated with Axis 1. Salinity was positively correlated with Axis 2(Table 4).The first axis separated the sand dominated bay station from the outer stations with finer sediments (clayey silt) (Fig 7). The second axis indicated the weak separation of the outer stations based on salinity. The relative length of the arrows representing the environmental variables indicated that sediment texture wasthe major influencing parameter. Affinity between the polychaete species and the environmental variables/stations is also represented in the triplot. The major polychaete species that were mostly associated with the sand dominated bay station were *Aonidellacirrobranchiata*, Capitellidae (gen. sp.), *Scoloplos armiger*, *S*. *uniramus*, *Eunice pennata*, *Cirratulus* sp., *Sabellastarte longa* and *Novafabriciabansei*. Dominant polychaete species that were typically present in the outer stations were *Paraprionospiopatiens*, *Paraprionospio*sp., *Aglaophamusdibranchis*, *Ninoe*sp.,*Heterospio*sp., *Magelonacincta*, *Cossuracoasta*, Maldanidae (gen. sp.) and *Sternaspisscutata*. *P*. *patiens* was the dominant species at the *Phyan* impacted outer stations (Fig 7).

**Table 3:** Spearman’s Rank correlation between univariate indices, trophic groups and environmental variables.

	S	*d*	*H’ (log2)*	*J’*	*ES*_(100)_	(1-λ′)	Macrophage	Microphage	Omnivore	Macrophage-Microphage	Macrophage-Omnivore
**Sand (%)**	0.47*	0.49*	0.43*		0.46*		0.49*		0.43*		0.42*
**Clay (%)**	-0.47*	-0.50*	-0.48*		-0.51**	-0.43*	-0.61***				-0.41*
**Corg (%)**									-0.52**		-0.48*
**Salinity (psu)**	0.58**	0.59**	0.59**	0.46*	0.63***	0.58**	0.74***			0.48*	0.43*
**pH**								-0.47*	-0.42*		

*p*< 0.05*

*p* < 0.01**

*p* < 0.001***

**Table 4:** Summary of the results of CCA analysis.

Environmental variables	Axis 1	Axis 2	Axis 3	Axis 4
Sand (%)	0.83***	0.27	-0.18	-0.07
Silt (%)	-0.82***	-0.27	0.16	0.09
Clay (%)	-0.63***	-0.18	0.26	-0.08
Corg (%)	-0.90***	0.13	0.22	0.03
DO (mg l^-1^)	-0.29	-0.11	-0.01	-0.71***
pH	-0.52**	-0.12	-0.29	-0.39*
Salinity (psu)	0.09	0.36*	-0.78***	-0.03
Eigenvalues	0.79	0.61	0.52	0.18
Species-environment correlations	0.97	0.90	0.87	0.76
Cumulative percentage variance				
of species data	21.4	37.8	51.9	56.8
of species-environment relation	35.4	62.7	86.0	94.2

*p*< 0.05*

*p* < 0.01**

*p* < 0.001***

## 4. Discussion

The present account provides the first ever diversity of polychaete species of the subtidal areas in and around the Malvan Marine Sanctuary,which could form the baseline for future ecological investigations. The only previous description is that of the intertidal marine fauna of Malvan described by Parulekar[30]. A dichotomy in the extent of cyclonic impacts on the four stations, based on their location, was apparent from the results. Rapid reduction in polychaete density, diversity indices and a concomitant increase in the dominance of the opportunistic species after *Phyan*,in the relatively more exposed three outer stations was indicative ofthe cyclonic impact in the study area. Decline in almost all indicators of the polychaete community structure atthe outer stations during Postmonsoon 2009 (*Phyan*) relative to Postmonsoon 2007 demonstrated the impact of *Phyan* over and above seasonal successional changes. These results are consistent with observations from post disturbance studies conducted elsewhere [3, 4]. In contrast, Hughes et al. [31] reported an increase in the species diversity in benthic assemblages of an intertidal mudflat along the Virginia coast, USA after the landfall of the Hurricane *Isabel* though, they had also reported higher density of opportunistic species and microphagous feeders post hurricane in line with our observations. As opposed to the outer stations, consistently healthy univariate indices at the bay station supports the viewthat the bay had withstood the onslaught of the cyclone and maintained a healthy benthic diversity.

Significant temporal variations in the polychaete assemblages between the pre-*Phyan* and the post-*Phyan*periods reinforced the conclusion of occurrence of major shifts in the polychaete community structure due to cyclone-induced instability of the substratum Improved diversity indices of the the last sampling occasion, Premonsoon 2012 and its temporal separation from the pre-*Phyan* sampling periods in the MDS plot implied that the recovery had occurred, albeit with an altered species composition.According to Chollett and Bone [32], during large-scale disturbances caused by meteorological events, major recolonization occurs through the introduction of larvae, juveniles and/or adults originating from farther localities leading to the establishment of new species composition. Posey et al. [10] have opined that the impacts on the composition of benthic communities fromepisodic storms were less than the background annual variability. Conversely, in our study, the polychaete communities had reduced diversity indices and an altered community composition immediately after *Phyan* as compared to previous analogous season data, implying severe and distinctcyclonic impact on polychaete community thanthe seasonal variations.

Feeding functional diversity has been widely used in many studies as an information tool complementary to the traditional diversity indices in an ecological impact assessment. Trophic diversity is presumed to be higher in undisturbed areas owing to the presence of superior species diversity [33]. On the contrary, perturbed sediments cause habitat alteration facilitating dominance of opportunistic species leading to reduced numbers of feeding types[34–35]. A shift in the feeding guild composition just after the passage of *Phyan* was observed. In the clayey-silt dominated outer stations, microphagous feeders were observed to have replaced other feeding types during postmonsoon and monsoon periods that may be the natural seasonal successional pattern in the study area. However, minor representation of diverse feeding types at the outer stations thatwere present before *Phyan*wasalmost entirely replaced by the microphages during Phyan 2009. Therefore, it is inferred that the occurrence of *Phyan* had resulted in complete dominance of microphages predominantly belonging to the Spionidaefamily. Spionidae are known to switch the feeding mode between suspension feeding and deposit feeding [24] depending on the background conditions. This adaptive mechanism probably facilitated the survival of spionids through the cyclone induced perturbations in their habitat. Similar increases in the density of microphage feeders as a direct effect of the cyclonic impact have been observed in other post impact studies [20, 31]. Fromthe post-*Phyan* observations,it was evident that the diverse feeding modes had made their re-appearance, thereby reducing the proportion of microphagous feeders signalingsubsequent system stabilization and progress of recovery. The re-establishment of macrophagy (carnivory) in the outer stations during post-*Phyan* years also indicated revivalof the polychaete community, as carnivores are generally more abundant in stable environments [36]. Relatively, the bay station had polychaetes with most diverse feeding strategies during Phyan 2009.Thus,the functional group approach revealedthat the physical stress due to *Phyan* was more severe in the outer stations while the bay station was the least impacted.

Dominance of fast growing opportunistic species in the aftermath of severe physical disturbances has been reported in analogous studies [3, 4, 20,31, 37]. Just after the cyclone (Phyan 2009), the opportunistic spionid, *P*.*patiens* entirely dominated the polychaete assemblages of the outer stations (86%) almost to the exclusion of other species. Predominance of opportunistic species after the disturbance is resultant of both passive advection and active immigration of opportunistic species and their larvae that thrive on the available space and food resources in the absence of sensitive species [32, 38]. Opportunistic characteristics of members of the Spionidae family like prolific reproduction with continuous supply of larvae, fast growth and early maturity enable rapid colonization of disturbed areas [38–39]. It was also apparent that the reappearance of equilibrium species had reduced thepredominance of *P*. *patiens*during the last two sampling periods, Postmonsoon 2011 and Premonsoon 2012, the former being the comparable post-disturbance season of Phyan2009.

The role of sediment as a major governing factor in influencing polychaete assemblages has been well established by past studies [40–41]. Coarse sediments provide interstitial spaces that present diverse habitats as well as variety of food sources for co-existence of multiple feeding groups [42–43] while finer sediments support predominantly microphage feeders [41,44]. This was also evident in the present investigation with the sandybay station (M4) harboring 55 polychaete species subscribing to the multiple feeding modes as against the outer stations (M1-M3), which weredominated by finer sediments and colonized by 44 polychaete species, most of which were microphagous feeders. An increase in the clay fraction of the sediments of the outer stations after monsoonal as well as cyclonic disturbance post *Phyan*was most likely due to the change in hydrodynamics associated with storms [45]. Correlation analyses indicated that the increase in clay fraction was followed by a decrease of polychaete community diversity and evenness values. Therefore, it may be possible that the disturbanceof bottom sediments led to textural changes that resulted in the shift in the community structure. Our results find resonance with Simboura et al. [46] who haveshownthat the natural factors such as intense hydrodynamic conditions led to a decline in the species diversity.

Critical velocity for erosion for silt is 1.6 m/s [47].Highercurrent velocities and enhanced turbulence in the outer region generated by the cyclone may have easily disturbed the bottom silty sediments the resultant sediment shift probably led to the disturbance to the existing speciose balanced polychaete community structure in outer stations. On the contrary, the critical erosion velocity for sand, which dominated the sediments at bay station M4, is relatively high as compared to silt particles and hence reworking of these sediments must have been to a lesser extent. It is also likely that the presence of the island off Malvan buffered the impacts of the physical disturbance on the benthic fauna at the bay station.

Thus the major impact of *Phyan* was a reduction inpolychaete species diversity and density coupled with the replacement of assemblages belonging to multiple functional groups with microphagous opportunistic species dominated polychaetegroups in the outer stations off Malvan. The recovery of the impacted polychaete communities was observed but with modified species and functional traits. Our observations concur with Texeidó et al. [6] who suggested that large occasional perturbances caused by cyclones could be potential drivers of benthic community dynamics leading to new species composition with altered functional groups. Present study also indicatedthat physical disturbances subsequent to cyclones do not have long term effects on macrofauna in areas largely free of human intervention. A perusal of the polychaete community parameters before and after the cyclonic turbulence implied good recovery potential of the area as suggested by Engle et al. [4]. Far reaching ecological impacts were absent at Malvan post *Phyan* and recovery seemed to be complete although with a different species composition. Post tsunami recovery of benthic community at Laem Son National Park was observed after 3 years [48]. On the contrary, the polychaete community in the Onagawa Bay was still recovering, three years after tsunami disturbance [20]. The ecological resurgence of benthic systems impacted by natural forces is reported to be faster in areas with little human interference [49] and therecovery potential of disturbed benthic assemblages is higher at marine protected areas vis-à-vis unprotected locations due to the acceleration in recolonization processes [50–51]. It, therefore, appears that Malvan presents a resilient environment and the revival potential of the system seems to be high given that it has a marine protected zone.

Results from observations on the real-time impacts of natural physical occurrences at different spatial and temporal scales will serve to aid better understanding of the ecological costs of such episodes and its recovery cycles [52]. To improve our forecast abilities of long-term community shifts, it is imperative to increase efforts in Studies similar to the one presented in this communication would undoubtedly contribute to the management and conservation initiatives of the coastal biodiversity of the Indian subcontinent. Predictive capabilities of responses of marine communities to future climate change are still at a nascent stage. Therefore, it is hoped that "incidental" studies such as the current investigation will augment efforts to create predictive modeling techniques applicable to complex benthic ecosystems.

## Supporting Information

S1 Table Polychaete species mean densities (ind.m^-2^) and standard deviations at Malvan during six sampling periods; Premonsoon 2007, Postmonsoon 2007, Phyan 2009, Monsoon 2011, Postmonsoon 2011 and Premonsoon 2012. (DOC)

## References

[1] Byju P, Prasanna Kumar S. Physical and biological response of the Arabian Sea to tropical cyclone Phyan and its implications. Mar Environ Res. 2011; 71: 325–330. 10.1016/j.marenvres.2011.02.008 21459432

[2] Flood PG, Jel JS. The effect of cyclone ‘David’ (January 1976) on the sediment distribution patterns on Heron Reef, Great Barrier Reef, Australia. Proceedings of third International Coral Reef Symposium. 1977; 2: 120–125.

[3] Balthis LA, Hyland JL, Bearden DW. Ecosystem responses to extreme natural events: impacts of three sequential hurricanes in Fall 1999 on sediment quality and condition of benthic fauna in the Neuse River Estuary, North Carolina. Environ Monit Assess. 2006; 119: 367–389. 16741819 10.1007/s10661-005-9031-6

[4] Engle VD, Hyland JL, Cooksey C. Effects of Hurricane Katrina on benthic macroinvertebrate communities along the northern Gulf of Mexico coast. Environ Monit Assess. 2009; 150: 193–209. 10.1007/s10661-008-0677-8 19052887

[5] Lomovasky BJ, Firstater FN, Salazar AG, Mendo J, Iribarne OO. Macro benthic community assemblage before and after the 2007 tsunami and earthquake at Paracas Bay Peru. J Sea Res. 2011; 65: 205–212.

[6] Teixido N, Casas E, Cebrián E, Linares C, Garrabou J. Impacts on coralligenous outcrop biodiversity of a dramatic coastal storm. PLoS One. 2013; 8: e53742 10.1371/journal.pone.0053742 23326496 PMC3542355

[7] Goldenberg SB, Landsea CW, Mestas-Nunez AM, Gray WM. The recent increase in Atlantic hurricane activity: causes and implications. Science. 2001; 293:474–479. 11463911 10.1126/science.1060040

[8] Elsner JB, Kossin JP, Jagger TH. The increasing intensity of the strongest tropical cyclones. Nature. 2008; 455: 92–95. 10.1038/nature07234 18769438

[9] Lucrezi S, Schlacher TA, Robinson W. Can storms and shore armouring exert additive effects on sandy-beach habitats and biota? Mar Freshwater Res. 2010; 61: 951–962.

[10] Posey M, Lindberg W, Alphin T, Vose F. Influence of storm disturbance on an offshore benthic community. B Mar Sci.1996; 59: 523–529.

[11] Joseph A, Prabhudesai RG, Mehra P, Sanil Kumar V, Radhakrishnan KV, Kumar V, et al Response of west Indian coastal regions and Kavaratti lagoon to the November -2009 tropical cyclone *Phyan*. Nat Hazards. 2011; 57: 293–312.

[12] Shiledar BAA, Khandagale PA, Singh VV. Impact of the cyclonic storm 'phyan' on marine fisheries along the Sindhudurg coast of Maharashtra. Marine Fisheries Information Service; Technical and Extension Series.2013; 215: 15–16.

[13] Wafar M, Venkataraman K, Ingole B, Ajmal Khan S, Lokabharathi P. State of Knowledge of Coastal and Marine Biodiversity of Indian Ocean Countries. PLoS One. 2011; 6: e14613 10.1371/journal.pone.0014613 21297949 PMC3031507

[14] Mukherjee N, Dahdouh-Geubas F, Kapoor V, Arthur R, Koedam N, Sridhar A, Shanker K. From Bathymetry to Bioshields: A review of post-Tsunami Ecological Research in India and its Implications for policy. Environ Manage. 2010; 46: 329–339. 10.1007/s00267-010-9523-1 20640420

[15] Kumar VK, Aboobacker VM, Saheed PP, Vethamony P. Coastal circulation along the central west coast of India during cyclone Phyan: measurements and numerical simulations. Nat Hazards. 2012; 64: 259–271.

[16] Doğan A, Çinar ME, Önen M, Ergen Z, Katağan T. Seasonal dynamics of soft bottom zoobenthic communities in polluted and unpolluted areas of Izmir Bay (Aegean Sea). Senckmarit. 2005; 35: 133–145.

[17] Won EJ, Raisuddin S, Shin KH. Evaluation of induction of metallothionein-like proteins (MTLPs) in the polychaetes for biomonitoring of heavy metal pollution in marine sediments. Mar Pollut Bull. 2008; 57: 544–551. 10.1016/j.marpolbul.2008.02.025 18395758

[18] Dean HK. The use of polychaetes (Annelida) as indicator species of marine pollution. Rev Biol Trop (Int. J. Trop. Biol.). 2008; 56:11–38.

[19] Musco l, Mikac B, Tataranni M, Giangrande A, Terlizzi A. The use of coarser taxonomy in the detection of long-term changes in polychaete assemblages. Mar Environ Res. 2011; 71: 131–138. 10.1016/j.marenvres.2010.12.004 21196046

[20] Abe H, Kobayashi G, Sato-Okoshi W. Impacts of the 2011 tsunami on the subtidalpolychaete assemblage and the following recolonization in Onagawa Bay, northeastern Japan. Mar Environ Res.2015; 112:86–95. 10.1016/j.marenvres.2015.09.011 26454517

[21] Grasshoff K, Ehrhardt M, Kremling K. Methods of seawater analysis (VerlagChemie); 1999. pp 1–419

[22] Buchanan JB. Sediment analysis In: Methods for study of marine benthos (second edition) Holme NA and McIntyre AD) Blackwell Scientific publications, Oxford and Edinburgh; 1984 pp 1–65.

[23] Walkey A, Black IA. An examination of the Degtjareff method for determining soil organic matter and a proposed modification of the chromic acid titration method. Soil Sci. 1934; 37: 28–30.

[24] Jumars PA, Dorgan KM, Lindsay SM. Diet of Worms Emended: An Update of Polychaete Feeding Guilds. Annu Rev Mar Sci. 2015; 7:497–520. 10.1146/annurev-marine-010814-020007 25251269

[25] Anderson MJ, Gorley RN, Clarke KR (2008) PERMANOVA+ for PRIMER:guide to software and statistical methods. Primer-e,Plymouth, UK.

[26] Hill MO, Gauch HG. Detrended correspondence analysis: an improved ordination technique. Vegetatio. 1980; 42: 47–58.

[27] terBraak CJF, Prentice IC. A theory of gradient analysis. AdvEcol Res. 1988; 18: 271–317.

[28] Härnström K, Karunasagar I, Godhe A. Phytoplankton species assemblages and their relationship to hydrographic factors-a study at the old port in Mangalore, coastal Arabian Sea. Indian J Mar Sci. 2009; 38: 224–234.

[29] terBraak CJF, Smilauer P (2002) CANOCO reference manual and user’s guide to Canoco for Windows: software for canonical community ordination (version 4.53). Microcomputer power, Ithaca, NY.

[30] Parulekar AH. Polychaetes from Maharashtra and Goa. J Bombay Nat Hist Soc. 1971; 68: 726–749

[31] Hughes C, Richardson CA, Luckenbach M, Seed R. Difficulties in separating hurricane induced effects from natural benthic succession: Hurricane Isabel, a case study from Eastern Virginia, USA. Estuar Coast Shelf S. 2009; 85: 377–386.

[32] Chollett I, Bone D. Effects of heavy rainfall on polychaetes: Differential spatial patterns generated by a large-scale disturbance. J Exp Mar Biol Ecol. 2007; 340: 113–125.

[33] Gamito S, Patricio J, Neto JM, Texeira H, Marques JC. Feeding diversity index as complementary information in the assessment of ecological quality status. Ecol Indic. 2012; 19:73–78.

[34] Gamito S, Furtado R. Feeding diversity in macroinvertebrate communities: A contribution to estimate the ecological status in shallow waters. Ecol Indic. 2009; 9:1009–1019.

[35] Peng S, Zhou R, Qin X, Shi H, Ding D. Application of macrobenthos functional groups to estimate the ecosystem health in a semi-enclosed bay. Mar Pollut Bull. 2013; 74: 302–310. 10.1016/j.marpolbul.2013.06.037 23849956

[36] Cheung SG, Lam NWY, Wu RSS, Shin PKS. Spatio-temporal changes of marine macrobenthic community in sub-tropical waters upon recovery from eutrophication. II.Life-history traits and feeding guilds of polychaete community. Mar Pollut Bull. 2008; 56: 297–307. 18061624 10.1016/j.marpolbul.2007.10.019

[37] Urabe J, Suzuki T, Nishita T, Makino W. Immediate ecological impacts of the 2011 Tohoku earthquake tsunami on intertidal flat communities. PLoS One. 2013; 8: e62779 10.1371/journal.pone.0062779 23650529 PMC3641098

[38] Savidge WB, Taghon GL. Passive and active components of colonization following two types of disturbance on intertidal sandflat. J Exp Mar Biol Ecol. 1988; 115:137–155.

[39] Grassle JF, Grassle JP. Opportunistic life histories and genetic systems in marine benthic polychaetes. J Mar Res. 1974; 32: 253–284.

[40] Labrune C, Gre´mare A, Amouroux JM, Sarda R, Gil J, Taboada S. Assessment of soft-bottom polychaete assemblages in the Gulf of Lions (NW Mediterranean) based on a mesoscale survey. Estuar Coast Shelf S. 2007; 71:133–147.

[41] Martins R, Sampaio L, Rodrigues AM, Quintino V. Soft-bottom Portuguese continental shelf polychaetes: Diversity and distribution. J Marine Syst. 2013; 123–124: 41–54.

[42] Carrasco F, Carbajal W. The Distribution of Polychaete Feeding Guilds in Organic Enriched Sediments of San Vicente Bay, Central Chile.Intemat Rev Hydrobiol.1998; 83: 233–249.

[43] Muniz P, Pires AMS. Trophic structure of polychaetes in the São Sebastião Channel (southeastern Brazil).Mar Biol. 1999; 134: 517–528.

[44] Del-Pilar-Ruso Y, De-la-Ossa Carretero JA, Loya-Fernández A, Ferrero-Vincente LM, Giménez-Casaldureo F, Sánchez-Lizaso JL. Assessment of soft-bottom polychaeta assemblage affected by a spatial confluence of impacts: Sewage and brine discharges. Mar Pollut Bull. 2009; 58: 765–786. 19349052 10.1016/j.marpolbul.2009.03.002

[45] Dellapenna TM, Kuehl SA, Schaffner LC. Ephemeral deposition, seabed mixing and fine-scale strata formation in the York River estuary, Chesapeake Bay.Estuar Coast Shelf S. 2003; 58: 621–643.

[46] Simboura N, Nicolaidou A, Thessalou-Legaki M. Polychaete communities of Greece: An Ecological Overview. P.S.Z.N. Mar Ecol. 2000; 21: 129–144.

[47] Rijn LC. 2007. Unified view of Sediment Transport by Currents and Waves. I: Initiation of Motion, Bed Roughness, and Bed-Load Transport. J Hydraul Eng. 2007; 133: 649–667.

[48] Kendall MA, Aryuthaka C, Chimonides J, Daungnamon D, Hills J, Jittanoon C, et al Post-Tsunami Recovery of Shallow Water Biota and Habitats on Thailand’s Andaman Coast. Polish J Environ Stud. 2009; 18: 69–75.

[49] Witmer AD, Roelke DL. Human interference prevents recovery of infaunal beach communities from hurricane disturbance. Ocean Coast Manage. 2014; 87: 52–60.

[50] Bevilacqua S, Terlizzi A, Fraschetti S, Giovanni GF, Boero F. Mitigating Human Disturbance: Can Protection Influence Trajectories of Recovery in Benthic Assemblages? J Anim Ecol. 2006; 75: 908–920. 17009754 10.1111/j.1365-2656.2006.01108.x

[51] Game ET, McDonald-Madden E, Puotinen ML, Possingham HP. Should We Protect the Strong or the Weak? Risk, Resilience, and the Selection of Marine Protected Areas.Conserv Biol. 22: 1619–1629. 10.1111/j.1523-1739.2008.01037.x 18759769

[52] Herkül K, Kotta J, Pärnoja M. Effect of physical disturbance on the soft sediment benthic macrophyte and invertebrate community in the northern Baltic Sea. Boreal Environ Res. 2011; 16: 209–219.

