# Supplementary material for: Polychaete Community of a Marine Protected Area along the West Coast of India—Prior and Post the Tropical Cyclone, Phyan
Source: PLoS One. 2016 Aug 24;11(8):e0159368. doi: 10.1371/journal.pone.0159368 (PMC4996641; doi:10.1371/journal.pone.0159368)
Supplement: S1 Table — (DOC) [file pone.0159368.s001.doc]

S1 Table: Polychaete species mean densities (ind.m-2) and standard deviations at Malvan during six sampling periods; Premonsoon 2007 , Postmonsoon 2007, Phyan 2009, Monsoon 2011 , Postmonsoon 2011 and Premonsoon 2012

|  |  |  |  |  |  |  |  |  |  |  |  |
| --- | --- | --- | --- | --- | --- | --- | --- | --- | --- | --- | --- |
| **Species population** | **M1** | **M2** | **M4** | **M1** | **M2** | **M3** | **M4** | **M1** | **M2** | **M3** | **M4** |
|  | **Pre-07** | **Pre-07** | **Pre-07** | **Pos-07** | **Pos-07** | **Pos-07** | **Pos-07** | **Phy-09** | **Phy-09** | **Phy-09** | **Phy-09** |
| Polynoidae (gen. sp.) | 0 | 0 | 0 | 0 | 0 | 0 | 19±13 | 0 | 0 | 0 | 13±25 |
| *Pholoe* sp. | 0 | 0 | 0 | 0 | 0 | 0 | 0 | 0 | 0 | 0 | 0 |
| *Eteone ornata* | 0 | 0 | 6±13 | 6±13 | 0 | 0 | 6±13 | 0 | 0 | 0 | 0 |
| *Phyllodoce capensis* | 0 | 0 | 0 | 0 | 0 | 0 | 0 | 0 | 0 | 0 | 0 |
| *Sigambra parva* | 6±13 | 0 | 6±13 | 0 | 0 | 0 | 0 | 0 | 0 | 0 | 0 |
| *Sigambra constricta* | 6±13 | 0 | 0 | 0 | 0 | 0 | 0 | 0 | 0 | 0 | 0 |
| Hesionidae (gen. sp.) | 0 | 0 | 0 | 0 | 0 | 0 | 0 | 0 | 0 | 0 | 0 |
| *Oxydromus* sp. | 0 | 0 | 0 | 0 | 0 | 0 | 0 | 0 | 0 | 0 | 0 |
| *Oxydromus spinosus* | 0 | 0 | 25±50 | 0 | 0 | 0 | 0 | 0 | 0 | 0 | 0 |
| *Oxydromus agilis* | 25±50 | 13±14 | 0 | 0 | 0 | 0 | 13±14 | 0 | 0 | 0 | 0 |
| *Oxydromus angustifrons* | 0 | 0 | 0 | 0 | 0 | 0 | 0 | 0 | 0 | 0 | 0 |
| *Leocrates claparedii* | 0 | 0 | 0 | 0 | 0 | 0 | 0 | 0 | 0 | 0 | 0 |
| *Syllis* sp. | 0 | 0 | 0 | 0 | 0 | 0 | 0 | 0 | 0 | 0 | 0 |
| *Syllis amica* | 0 | 0 | 0 | 0 | 0 | 0 | 0 | 0 | 0 | 0 | 6±13 |
| Nereididae (gen. sp.) | 25±50 | 0 | 19±38 | 19±24 | 13±14 | 0 | 6±13 | 0 | 0 | 0 | 6±13 |
| *Aglaophamus dibranchis* | 50±35 | 13±25 | 0 | 13±14 | 0 | 0 | 0 | 0 | 0 | 0 | 31±13 |
| *Micronephthys oculifera* | 0 | 0 | 0 | 0 | 0 | 0 | 0 | 0 | 0 | 0 | 0 |
| Glyceridae (gen. sp.) | 0 | 0 | 25±50 | 0 | 0 | 0 | 0 | 0 | 0 | 0 | 0 |
| *Glycera longipinnis* | 0 | 6±13 | 119±85 | 13±25 | 19±24 | 0 | 31±38 | 0 | 0 | 0 | 31±47 |
| *Glycera alba* | 0 | 0 | 0 | 0 | 0 | 0 | 6±13 | 0 | 0 | 0 | 0 |
| Eunicidae (gen. sp.) | 0 | 6±13 | 31±47 | 0 | 0 | 0 | 0 | 0 | 0 | 6±13 | 0 |
| *Eunice* sp. | 0 | 0 | 0 | 0 | 0 | 0 | 0 | 0 | 0 | 0 | 0 |
| *Eunice pennata* | 0 | 0 | 169±182 | 0 | 13±25 | 0 | 75±87 | 0 | 0 | 0 | 13±25 |
| *Lysidice* sp*.* | 0 | 0 | 25±50 | 0 | 0 | 0 | 0 | 0 | 0 | 0 | 0 |
| *Lysidice ninetta* | 0 | 0 | 0 | 0 | 0 | 0 | 19±24 | 0 | 0 | 0 | 0 |
| *Diopatra* sp. | 0 | 0 | 0 | 0 | 0 | 0 | 0 | 0 | 0 | 0 | 0 |
| *Diopatra cuprea* | 0 | 0 | 6±13 | 0 | 0 | 0 | 19±24 | 0 | 0 | 0 | 13±25 |
| *Onuphis quinquedens* | 0 | 0 | 13±25 | 0 | 0 | 0 | 0 | 0 | 0 | 0 | 6±13 |
| *Nematonereis* sp*.* | 0 | 0 | 0 | 0 | 0 | 0 | 0 | 0 | 0 | 0 | 0 |
| *Ninoe* sp. | 0 | 0 | 0 | 0 | 0 | 0 | 0 | 0 | 0 | 0 | 0 |
| *Ninoe lagosiana* | 19±24 | 6±13 | 6±13 | 0 | 0 | 0 | 0 | 0 | 0 | 0 | 0 |
| *Lumbrineris* sp*.* | 0 | 25±50 | 38±43 | 6±13 | 6±13 | 0 | 0 | 13±14 | 6±13 | 0 | 25±29 |
| *Lumbrineris meteorana* | 0 | 0 | 0 | 0 | 0 | 0 | 0 | 0 | 0 | 0 | 0 |
| *Lumbrineris latreilli* | 6±13 | 0 | 0 | 0 | 0 | 0 | 0 | 0 | 0 | 0 | 0 |
| *Lumbrineris brevicirra* | 0 | 0 | 0 | 0 | 0 | 0 | 0 | 0 | 0 | 0 | 0 |
| *Schistomeringos rudolphi* | 0 | 0 | 0 | 0 | 0 | 0 | 0 | 0 | 0 | 0 | 0 |
| Spionidae (gen. sp.) | 13±14 | 0 | 0 | 94±188 | 0 | 0 | 44±88 | 0 | 0 | 0 | 0 |
| *Boccardia polybranchia* | 0 | 0 | 131±213 | 0 | 0 | 0 | 6±14 | 0 | 0 | 0 | 0 |
| *Aonidella cirrobranchiata* | 0 | 0 | 44±59 | 0 | 69±76 | 6±13 | 119±140 | 19±24 | 0 | 0 | 144±238 |
| *Paraprionospio* sp*.* | 0 | 0 | 0 | 0 | 0 | 0 | 0 | 0 | 0 | 0 | 0 |
| *Paraprionospio patiens* | 0 | 13±14 | 69±121 | 3025±585 | 1606±1164 | 913±660 | 0 | 869±288 | 438±249 | 375±347 | 313±336 |
| *Heterospio* sp. | 0 | 0 | 0 | 0 | 0 | 0 | 0 | 0 | 0 | 0 | 0 |
| *Magelona cincta* | 6±13 | 0 | 19±13 | 869±652 | 181±52 | 63±66 | 0 | 0 | 0 | 0 | 25±50 |
| *Magelona pulchella* | 0 | 0 | 0 | 0 | 0 | 0 | 0 | 0 | 0 | 0 | 0 |
| Cirratulidae (gen. sp.) | 0 | 0 | 50±100 | 0 | 0 | 0 | 0 | 0 | 0 | 0 | 0 |
| *Cirratulus* sp. | 0 | 0 | 269±103 | 0 | 0 | 0 | 100±98 | 0 | 0 | 0 | 25±58 |
| *Cirratulus africanus* | 0 | 6±13 | 0 | 0 | 0 | 0 | 0 | 0 | 0 | 0 | 0 |
| *Aphelochaeta filiformis* | 0 | 0 | 0 | 0 | 0 | 0 | 0 | 0 | 0 | 0 | 0 |
| *Poecilochaetus serpens* | 0 | 0 | 0 | 0 | 6 | 13±25 | 0 | 0 | 0 | 0 | 0 |
| *Trochochaeta* sp. | 0 | 0 | 0 | 0 | 0 | 0 | 0 | 0 | 0 | 0 | 0 |
| Orbiniidae (gen. sp.) | 0 | 0 | 144±288 | 0 | 0 | 0 | 0 | 0 | 0 | 0 | 0 |
| *Scoloplos uniramus* | 0 | 0 | 0 | 0 | 0 | 0 | 0 | 0 | 0 | 0 | 0 |
| *Scoloplos armiger* | 0 | 0 | 700±684 | 0 | 0 | 0 | 150±129 | 0 | 0 | 0 | 150±235 |
| *Armandia* sp. | 0 | 0 | 0 | 0 | 0 | 0 | 6±13 | 0 | 0 | 0 | 0 |
| *Cossura coasta* | 94±13 | 213±25 | 6±13 | 63±43 | 113±25 | 125±170 | 0 | 25±35 | 169±66 | 31±13 | 0 |
| *Cossura longocirrata* | 0 | 0 | 0 | 0 | 0 | 0 | 0 | 0 | 0 | 0 | 0 |
| Capitellidae (gen. sp.) | 6±13 | 250±467 | 138±1643 | 0 | 0 | 6±13 | 13±14 | 0 | 0 | 0 | 6±13 |
| *Mediomastus* sp. | 0 | 206±178 | 0 | 0 | 0 | 0 | 0 | 0 | 0 | 0 | 0 |
| *Notomastus* sp. | 6±13 | 19±38 | 0 | 0 | 0 | 0 | 0 | 0 | 0 | 0 | 6 |
| *Parheteromastus* sp. | 0 | 125±203 | 119±238 | 0 | 0 | 0 | 0 | 0 | 0 | 0 | 0 |
| *Capitella capitata* | 175±84 | 50±61 | 0 | 0 | 0 | 0 | 0 | 0 | 0 | 0 | 0 |
| Maldanidae (gen. sp.) | 0 | 0 | 0 | 0 | 0 | 0 | 0 | 0 | 0 | 0 | 0 |
| *Sternaspis scutata* | 0 | 0 | 0 | 0 | 0 | 0 | 0 | 0 | 0 | 0 | 0 |
| *Pherusa* sp. | 0 | 0 | 0 | 0 | 0 | 0 | 13±25 | 0 | 0 | 0 | 0 |
| Sabellariidae (gen. sp.) | 0 | 0 | 0 | 0 | 0 | 0 | 13±25 | 0 | 0 | 0 | 44±59 |
| *Isolda pulchella* | 0 | 0 | 0 | 0 | 0 | 0 | 0 | 0 | 0 | 0 | 0 |
| Terebellidae (gen. sp.) | 0 | 0 | 0 | 0 | 0 | 0 | 13±25 | 0 | 0 | 0 | 6±13 |
| *Loimia batilla* | 0 | 0 | 0 | 0 | 0 | 0 | 0 | 0 | 0 | 0 | 0 |
| Sabellidae (gen. sp.) | 0 | 0 | 0 | 0 | 0 | 0 | 0 | 0 | 0 | 0 | 0 |
| *Sabellastarte longa* | 0 | 0 | 500±639 | 0 | 0 | 0 | 50±41 | 0 | 0 | 0 | 0 |
| *Novafabricia bansei* | 0 | 0 | 281±273 | 0 | 0 | 0 | 88±66 | 0 | 0 | 0 | 0 |
| *Spirobranchus kraussii* | 0 | 0 | 0 | 0 | 0 | 0 | 6±13 | 0 | 0 | 0 | 0 |
| *Hydroides homoceros* | 0 | 0 | 0 | 0 | 0 | 0 | 0 | 0 | 0 | 0 | 25±71 |

| S1 Table: Contd. |  |  |  |  |  |  |  |  |  |  |  |  |
| --- | --- | --- | --- | --- | --- | --- | --- | --- | --- | --- | --- | --- |
| **Species population** | **M1** | **M2** | **M3** | **M4** | **M1** | **M2** | **M3** | **M4** | **M1** | **M2** | **M3** | **M4** |
|  | **Mon-11** | **Mon-11** | **Mon-11** | **Mon-11** | **Pos-11** | **Pos-11** | **Pos-11** | **Pos-11** | **Pre-12** | **Pre-12** | **Pre-12** | **Pre-12** |
| Polynoidae (gen. sp.) | 0 | 0 | 0 | 0 | 0 | 0 | 0 | 0 | 0 | 0 | 0 | 6±13 |
| *Pholoe* sp. | 0 | 0 | 0 | 0 | 0 | 0 | 0 | 6±13 | 0 | 0 | 0 | 0 |
| *Eteone ornata* | 0 | 0 | 0 | 0 | 0 | 0 | 0 | 0 | 0 | 0 | 0 | 0 |
| *Phyllodoce capensis* | 0 | 0 | 0 | 0 | 0 | 0 | 0 | 0 | 6±13 | 0 | 0 | 31±47 |
| *Sigambra parva* | 0 | 0 | 0 | 6±13 | 6±13 | 0 | 0 | 6±13 | 19±24 | 19±24 | 6±13 | 0 |
| *Sigambra constricta* | 0 | 0 | 0 | 0 | 0 | 0 | 0 | 0 | 0 | 0 | 0 | 0 |
| Hesionidae (gen. sp.) | 0 | 0 | 0 | 0 | 0 | 0 | 0 | 0 | 6.25 | 0 | 0 | 0 |
| *Oxydromus* sp. | 0 | 0 | 13±25 | 0 | 0 | 0 | 0 | 0 | 0 | 6±13 | 0 | 0 |
| *Oxydromus spinosus* | 0 | 0 | 0 | 0 | 0 | 0 | 0 | 0 | 0 | 0 | 0 | 0 |
| *Oxydromus agilis* | 0 | 0 | 0 | 0 | 0 | 0 | 0 | 0 | 0 | 0 | 0 | 0 |
| *Oxydromus angustifrons* | 0 | 6±13 | 0 | 0 | 0 | 0 | 0 | 0 | 0 | 0 | 0 | 0 |
| *Leocrates claparedii* | 0 | 0 | 0 | 0 | 0 | 0 | 0 | 0 | 0 | 0 | 0 | 6±13 |
| *Syllis* sp. | 0 | 0 | 0 | 0 | 0 | 0 | 0 | 0 | 0 | 0 | 0 | 19±13 |
| *Syllis amica* | 0 | 0 | 0 | 0 | 0 | 0 | 0 | 0 | 0 | 0 | 0 | 0 |
| Nereididae (gen. sp.) | 0 | 0 | 0 | 0 | 0 | 0 | 0 | 6±13 | 0 | 0 | 0 | 0 |
| *Aglaophamus dibranchis* | 0 | 0 | 0 | 0 | 25±29 | 31±47 | 6±13 | 106±116 | 56±52 | 38±32 | 6±13 | 13±25 |
| *Micronephthys oculifera* | 0 | 0 | 0 | 0 | 0 | 0 | 0 | 0 | 0 | 0 | 0 | 38±60 |
| Glyceridae (gen. sp.) | 0 | 0 | 0 | 0 | 0 | 0 | 0 | 0 | 0 | 0 | 0 | 0 |
| *Glycera longipinnis* | 0 | 0 | 0 | 0 | 0 | 0 | 0 | 0 | 0 | 0 | 0 | 0 |
| *Glycera alba* | 0 | 0 | 0 | 0 | 0 | 0 | 0 | 94±52 | 13±14 | 0 | 0 | 6±13 |
| Eunicidae (gen. sp.) | 0 | 0 | 0 | 0 | 0 | 0 | 13±25 | 0 | 0 | 0 | 0 | 0 |
| *Eunice* sp. | 0 | 0 | 0 | 0 | 0 | 0 | 0 | 13±14 | 0 | 0 | 0 | 213±113 |
| *Eunice pennata* | 0 | 0 | 0 | 0 | 0 | 0 | 0 | 0 | 0 | 0 | 0 | 0 |
| *Lysidice* sp. | 0 | 0 | 0 | 0 | 0 | 0 | 0 | 0 | 0 | 0 | 0 | 31±63 |
| *Lysidice ninetta* | 0 | 0 | 0 | 0 | 0 | 0 | 0 | 0 | 0 | 0 | 0 | 0 |
| *Diopatra* sp. | 0 | 0 | 0 | 0 | 0 | 0 | 0 | 13±14 | 0 | 13±25 | 0 | 0 |
| *Diopatra cuprea* | 0 | 0 | 0 | 0 | 0 | 0 | 0 | 0 | 0 | 0 | 0 | 0 |
| *Onuphis quinquedens* | 0 | 0 | 0 | 0 | 0 | 0 | 0 | 0 | 0 | 0 | 0 | 0 |
| *Nematonereis* sp*.* | 0 | 0 | 0 | 0 | 0 | 0 | 0 | 0 | 0 | 0 | 0 | 81±31 |
| *Ninoe* sp. | 0 | 25±20 | 0 | 0 | 0 | 0 | 0 | 0 | 156±24 | 225±41 | 169±63 | 0 |
| *Ninoe lagosiana* | 0 | 0 | 0 | 0 | 0 | 0 | 0 | 0 | 0 | 0 | 0 | 0 |
| *Lumbrineris* sp. | 13±14 | 0 | 0 | 0 | 0 | 0 | 0 | 19±24 | 0 | 0 | 0 | 6±13 |
| *Lumbrineris meteorana* | 0 | 13±14 | 13±14 | 0 | 25±29 | 0 | 0 | 0 | 0 | 0 | 0 | 0 |
| *Lumbrineris latreilli* | 0 | 0 | 0 | 0 | 0 | 0 | 0 | 0 | 0 | 0 | 0 | 0 |
| *Lumbrineris brevicirra* | 0 | 0 | 0 | 0 | 0 | 0 | 13±14 | 0 | 0 | 0 | 0 | 0 |
| *Schistomeringos rudolphi* | 0 | 0 | 0 | 0 | 0 | 0 | 0 | 0 | 0 | 0 | 0 | 6±13 |
| Spionidae (gen. sp.) | 0 | 0 | 0 | 0 | 0 | 0 | 0 | 0 | 0 | 0 | 0 | 0 |
| *Boccardia polybranchia* | 0 | 0 | 0 | 0 | 0 | 0 | 0 | 0 | 0 | 0 | 0 | 0 |
| *Aonidella cirrobranchiata* | 0 | 0 | 6±13 | 0 | 0 | 0 | 0 | 63±85 | 0 | 6±13 | 0 | 6±13 |
| *Paraprionospio* sp. | 1344±858 | 344±232 | 63 | 0 | 44±24 | 38±43 | 19±24 | 0 | 13±14 | 6±13 | 0 | 0 |
| *Paraprionospio patiens* | 0 | 0 | 0 | 13±25 | 0 | 0 | 0 | 1538±1449 | 0 | 0 | 0 | 0 |
| *Heterospio* sp. | 0 | 75±20 | 13±14 | 0 | 0 | 0 | 0 | 0 | 169±113 | 238±180 | 188±123 | 0 |
| *Magelona cincta* | 0 | 6±13 | 0 | 0 | 0 | 13±14 | 75±74 | 181±213 | 63±92 | 63±48 | 13±14 | 0 |
| *Magelona pulchella* | 0 | 0 | 0 | 0 | 50±29 | 0 | 31±63 | 25±35 | 0 | 0 | 0 | 0 |
| Cirratulidae (gen. sp.) | 0 | 0 | 0 | 0 | 13±25 | 0 | 6±13 | 119±177 | 0 | 0 | 25±20 | 13±14 |
| *Cirratulus* sp. | 0 | 6±13 | 0 | 0 | 0 | 0 | 0 | 0 | 0 | 0 | 0 | 0 |
| *Cirratulus africanus* | 0 | 0 | 0 | 0 | 0 | 0 | 0 | 0 | 0 | 0 | 0 | 0 |
| *Aphelochaeta filiformis* | 0 | 0 | 6±13 | 0 | 0 | 0 | 0 | 0 | 119±31 | 38±14 | 0 | 13±25 |
| *Poecilochaetus serpens* | 0 | 0 | 0 | 0 | 0 | 0 | 0 | 0 | 0 | 0 | 0 | 0 |
| *Trochochaeta* sp. | 0 | 0 | 0 | 0 | 0 | 0 | 0 | 0 | 0 | 6±13 | 0 | 13±25 |
| Orbiniidae (gen. sp.) | 0 | 0 | 0 | 0 | 0 | 0 | 0 | 0 | 0 | 0 | 0 | 0 |
| *Scoloplos uniramus* | 0 | 0 | 0 | 25±29 | 0 | 0 | 0 | 394±397 | 0 | 0 | 0 | 406±433 |
| *Scoloplos armiger* | 0 | 0 | 0 | 0 | 0 | 0 | 0 | 0 | 0 | 0 | 0 | 0 |
| *Armandia* sp. | 0 | 0 | 0 | 0 | 0 | 0 | 0 | 0 | 0 | 0 | 0 | 38±32 |
| *Cossura coasta* | 88±72 | 600±256 | 88±72 | 6±13 | 419±126 | 38±32 | 825±423 | 6±13 | 131±55 | 619±103 | 425±212 | 0 |
| *Cossura longocirrata* | 0 | 0 | 0 | 0 | 25±50 | 0 | 0 | 0 | 0 | 0 | 0 | 0 |
| Capitellidae (gen. sp.) | 0 | 0 | 6±13 | 94±188 | 31±38 | 0 | 0 | 413±497 | 0 | 0 | 0 | 44±72 |
| *Mediomastus* sp. | 56±31 | 0 | 0 | 0 | 0 | 0 | 0 | 0 | 0 | 0 | 0 | 0 |
| *Notomastus* sp. | 0 | 0 | 0 | 0 | 0 | 0 | 0 | 0 | 13±14 | 6±13 | 0 | 0 |
| *Parheteromastus* sp. | 0 | 0 | 0 | 0 | 0 | 0 | 0 | 0 | 0 | 0 | 0 | 0 |
| *Capitella capitata* | 0 | 0 | 0 | 0 | 0 | 0 | 0 | 0 | 0 | 0 | 0 | 0 |
| Maldanidae (gen. sp.) | 0 | 0 | 19±24 | 0 | 0 | 0 | 0 | 0 | 181±24 | 388±113 | 119±66 | 0 |
| *Sternaspis scutata* | 0 | 13±14 | 0 | 0 | 0 | 0 | 0 | 0 | 6±13 | 531±380 | 19±38 | 0 |
| *Pherusa* sp. | 0 | 0 | 0 | 0 | 0 | 0 | 0 | 0 | 0 | 0 | 0 | 0 |
| Sabellariidae (gen. sp.) | 0 | 0 | 0 | 0 | 0 | 0 | 0 | 0 | 0 | 0 | 0 | 6±13 |
| *Isolda pulchella* | 0 | 0 | 0 | 0 | 0 | 0 | 0 | 6±13 | 0 | 0 | 0 | 63±60 |
| Terebellidae (gen. sp.) | 0 | 0 | 0 | 0 | 0 | 0 | 0 | 0 | 0 | 0 | 0 | 13±25 |
| *Loimia batilla* | 0 | 0 | 0 | 0 | 0 | 0 | 0 | 0 | 6±13 | 0 | 0 | 0 |
| Sabellidae (gen. sp.) | 0 | 0 | 0 | 0 | 0 | 0 | 0 | 0 | 0 | 0 | 0 | 25±20 |
| *Sabellastarte longa* | 0 | 0 | 0 | 0 | 0 | 0 | 0 | 0 | 0 | 0 | 0 | 0 |
| *Novafabricia bansei* | 0 | 0 | 0 | 0 | 0 | 0 | 0 | 0 | 0 | 0 | 0 | 0 |
| *Spirobranchus kraussii* | 0 | 0 | 0 | 0 | 0 | 0 | 0 | 0 | 0 | 0 | 0 | 0 |
| *Hydroides homoceros* | 0 | 0 | 0 | 0 | 0 | 0 | 0 | 0 | 0 | 0 | 0 | 0 |
